# Sickle-Cell Trait as a Risk Factor for an Unprovoked Venous Thromboembolism: A Case Report

**DOI:** 10.7759/cureus.51142

**Published:** 2023-12-26

**Authors:** James D Cross, Brendan P Mackey, Umme Yasmin

**Affiliations:** 1 Family Medicine, Drexel University College of Medicine, Philadelphia, USA; 2 Family Medicine, Kaiser Permanente, Glen Burnie, USA

**Keywords:** sickle cell complications, deep vein thrombosis (dvt), risk assessment tools, sickle cell trait, venous thromboembolism (vte), pe

## Abstract

In this case report, we examine the increased risk of venous thromboembolism (VTE) in patients with sickle-cell trait (SCT), illustrated by a patient with SCT who developed pulmonary embolism (PE) despite low scores on conventional risk assessment tools. The case prompts both a discussion of risk assessment and management strategies in this population.

## Introduction

Sickle-cell trait (SCT) is a state in which an individual possesses a single mutated allele for hemoglobin S (HbS) [1]. It is distinct from sickle-cell disease (SCD), in which both alleles are mutated. Although SCD is known for clinical complications such as pain crises and anemia, individuals with SCT remain predominantly asymptomatic [1-3].

In contrast to its reputation as a benign condition, recent studies have explored a potential association between SCT and an increased risk of venous thromboembolism (VTE), which encompasses conditions such as deep vein thrombosis (DVT) and pulmonary embolism (PE) [4-7]. The proposed mechanisms for this association include vessel occlusion, blood stasis, and continuous activation of the coagulation cascade, but the exact process has not been confirmed [8,9].

In support of this proposed connection, we present the case of a 44-year-old male with SCT and obesity with no other risk factors, who developed PE despite low pretest probability on several conventional risk stratification tools.

## Case presentation

A 44-year-old African American male presented to the clinic after experiencing three days of shortness of breath and chest pain on inspiration. Initially, the patient woke up with chest pressure and right-sided stabbing pain that worsened upon inspiration and radiated to his back. He reported difficulty in climbing stairs and breathlessness during basic activities. His symptoms improved over the next three days with only mild pain upon deep inspiration and minimal shortness of breath at the time of presentation.

The patient, an active real estate agent, reported no history of similar symptoms or periods of prolonged immobility. He had a medical history significant only for obesity (BMI 38.5), hyperlipidemia, and being a carrier of SCT.

**Figure 1:**
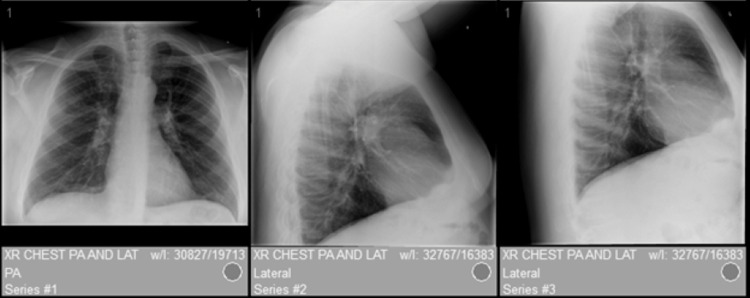
Chest X-rays showing no pathology.

**Figure 2:**
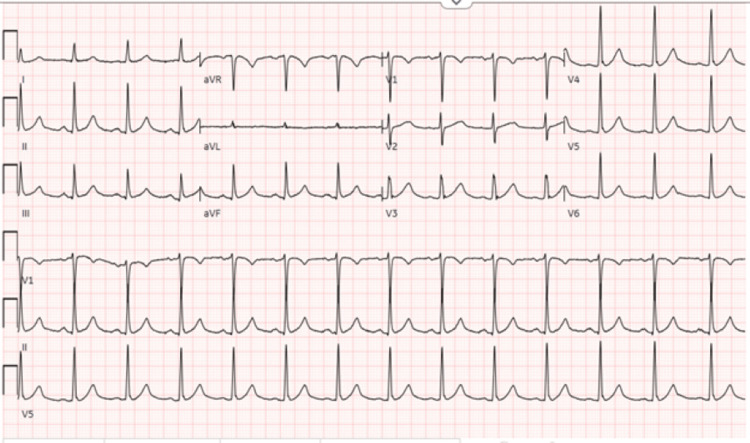
Electrocardiogram showing normal sinus rhythm.

Vitals were taken in the office (Table 1). Of note, his in-office SpO_2_ was 95%. Physical examination was unrevealing including negative Homan sign. Chest X-ray (Figure 1) and electrocardiogram (Figure 2) were non-contributory. Laboratory tests showed a normal complete blood count, comprehensive metabolic panel, and elevated D-dimer (Tables 2-3).

**Table 1:** Vitals

Measurement	Value
BP	131/82
Pulse	90
Temp	97.1 °F (36.2 °C) (Oral)
Resp	16
Ht	6' 8" (2.032 m)
Wt	350 lb (158.8 kg)
SpO_2_	95%
BMI	38.45 kg/m²

**Table 2:** Complete blood count (CBC) with differential Hgb: hemoglobin, MCH: mean corpuscular hemoglobin, MCHC: mean corpuscular hemoglobin concentration, MCV: mean corpuscular volume, RBC: red blood cell, RDW: red cell distribution width, WBC: white blood cells

Parameter	Patient Value	Reference Range
Hematocrit	42	41%-53%
Hgb	13.9	13.5-17.5 g/dL
MCH	27.5	25.4-34.6 pg/cell
MCHC	33.1	31%-36% Hb/cell
MCV	83	80-100 µm^3^
Platelets	270	182-396 K/mm^3^
RBC, Auto	5.06	4.70-6.10 M/uL
RDW, Blood	12.6	11.6%-14.8%
WBC's Auto	9.3	3.98-10.04K/mm^3^
Neutrophils %	50.6	50.0%-70.0%
Lymphocytes	3.46	1.00-4.30 K/uL
Monos %	10.2	0.0%-15.0%
Monocytes	0.95	0.00-1.10 K/uL
Eosinophils %	1.3	0.0%-06.0%
Eosinophils	0.12	0.00-0.60 K/uL
Basophils %	0.5	0.0%-2.0%
Basophils	0.05	0.00-0.20 K/uL
Neutrophils	4.72	2.40-7.60 K/uL

**Table 3:** CMP and D-dimer values CMP: comprehensive metabolic panel, BUN: blood urea nitrogen, eGFR: estimated glomerular filtration rate, ALT: alanine aminotransferase, AST: aspartate aminotransferase, EIA: enzyme immunoassay

Parameter	Patient Value	Reference Range
BUN	15	6-24 mg/dL
Creatinine	1	0.76-1.27 mg/dL
Sodium	142	134-144 mmol/L
Potassium	4.9	3.5-5.2 mmol/L
Chloride	104	96-106 mmol/L
CO_2_	26	20-29 mmol/L
eGFR	>60	>59 mL/min/1.73
Calcium	9.7	8.7-10.2 mg/dL
Total Protein	7.1	6.0-8.5 g/dL
Alkaline Phosphatase	63	39-117 IU/L
Globulin	2.3	1.5-4.5 g/dL
ALT	7	0-44 IU/L
AST	10	0-40 IU/L
Bilirubin, Total	0.3	0.0-1.2 mg/dL
Albumin	4.8	3.5-5.5 g/dL
D-DIMER, EIA	2.37 (H)	0-0.50 mg/L

**Figure 3:**
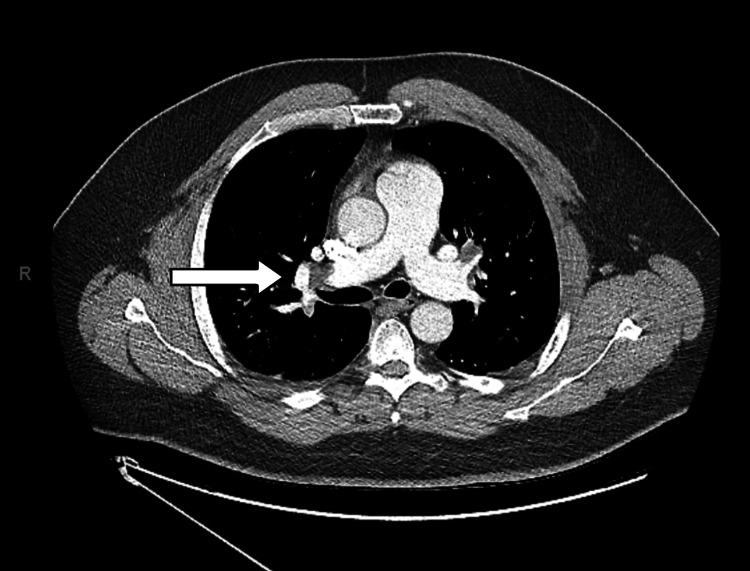
Computed tomography angiography (CTA) showing pulmonary embolism in the distal right pulmonary artery.

Given the low pretest probability based on the Wells and Geneva scores and having met pulmonary embolism rule-out criteria (PERC), initial assessments suggested a less than 1.5% risk for PE. Although this was the case his elevated D-dimer and shortness of breath prompted urgent computed tomography angiography (CTA) which identified extensive pulmonary emboli in the distal right pulmonary artery and the segmental and subsegmental pulmonary arteries supplying all five lobes, mild consolidative opacity in the right middle lobe (Figure 3).

The patient was immediately started on rivaroxaban 15 mg two times a day and scheduled for an echocardiogram (ECHO) in three months to assess for pulmonary hypertension and low dose CT chest to follow up right middle lobe opacity. Given the patient’s age, absence of clear provoking risk factors, and high risk of recurrent VTE, indefinite anticoagulation was recommended.

## Discussion

In a population-based cohort study of 30,424 individuals, Little et al. demonstrated a higher incidence of VTE in 6,758 SCT carriers compared to non-carriers [4]. Notably, this association held even after accounting for variables like body mass index (BMI), which was the sole other risk factor of note in our case presentation. Given these findings and the contributions of Naik and Noubiap, which further emphasize an increased risk of PE in particular, we provide support for a possible inherent thrombotic risk associated with SCT in our case presentation [5,10].

As both our case presentation and recent research support a possible link, we favor a need for increased surveillance of these patients. In the event that genetic predisposition does contribute to PE, clinical scoring systems such as the Modified Wells, Revised Geneva, and PERC become of lesser use and potentially enable these patients to evade detection [11,12]. As seen in our case presentation, the patient exhibited <1.5% risk in these metrics and was not recommended to receive further workup based on PERC scores. This emphasizes the need for a more comprehensive approach to assessing PE risk in SCT carriers as the existing models, while robust, do not account for inherited risks.

In addition to risk assessment concerns, our case raises some questions about approaches to management. In patients with first-time unprovoked PE, indefinite anticoagulation is in line with current recommendations for those with persistent risk factors and low bleeding risk [13,14]. At this time, SCT is not considered a persistent risk factor but raises the question of whether or not it should be in the context of recent research. In which case, indefinite treatment should be discussed. Alternative options have been debated such as the use of Disabilities of the Arm, Shoulder and Hand (DASH) scores to further assess an annual risk of PE recurrence [13-16]. For example, a DASH score ≤1, which indicates an annual PE risk of 3.1%, may lead some clinicians to consider discontinuing anticoagulation while continuing D-dimer monitoring [16]. This is an approach that could be relevant for SCT patients because it provides an assessment that remains uninfluenced by both inherent and modifiable risk factors.

## Conclusions

This case report highlights the limitations of current risk assessment strategies for patients with SCT and provides comments on approaches to both VTE prevention and management. Given that recent research has demonstrated an association with an increased risk of unprovoked VTE, there may now be a need to reason beyond current risk assessment tools and consider additional criteria that may enhance predictive capabilities in these patients.

## References

[1] Pauling L, Itano HA, Singer SJ (1949). Sickle cell anemia a molecular disease. Science.

[2] Rees DC, Williams TN, Gladwin MT (2010). Sickle cell disease. Lancet.

[3] Tsaras G, Owusu-Ansah A, Boateng FO, Amoateng-Adjepong Y (2009). Complications associated with sickle cell trait: a brief narrative review. Am J Med.

[4] Little I, Vinogradova Y, Orton E, Kai J, Qureshi N (2017). Venous thromboembolism in adults screened for sickle cell trait: a population-based cohort study with nested case-control analysis. BMJ Open.

[5] Naik RP, Streiff MB, Haywood C Jr, Nelson JA, Lanzkron S (2013). Venous thromboembolism in adults with sickle cell disease: a serious and under-recognized complication. Am J Med.

[6] Yu TT, Nelson J, Streiff MB, Lanzkron S, Naik RP (2016). Risk factors for venous thromboembolism in adults with hemoglobin SC or Sβ(+) thalassemia genotypes. Thromb Res.

[7] Seaman CD, Yabes J, Li J, Moore CG, Ragni MV (2014). Venous thromboembolism in pregnant women with sickle cell disease: a retrospective database analysis. Thromb Res.

[8] Austin H, Key NS, Benson JM, Lally C, Dowling NF, Whitsett C, Hooper WC (2007). Sickle cell trait and the risk of venous thromboembolism among blacks. Blood.

[9] Naik RP, Streiff MB, Lanzkron S (2013). Sickle cell disease and venous thromboembolism: what the anticoagulation expert needs to know. J Thromb Thrombolysis.

[10] Noubiap JJ, Temgoua MN, Tankeu R, Tochie JN, Wonkam A, Bigna JJ (2018). Sickle cell disease, sickle trait and the risk for venous thromboembolism: a systematic review and meta-analysis. Thromb J.

[11] Klok FA, Mos IC, Nijkeuter M, Righini M, Perrier A, Le Gal G, Huisman MV (2008). Simplification of the revised Geneva score for assessing clinical probability of pulmonary embolism. Arch Intern Med.

[12] Douma RA, Gibson NS, Gerdes VE, Büller HR, Wells PS, Perrier A, Le Gal G (2009). Validity and clinical utility of the simplified Wells rule for assessing clinical probability for the exclusion of pulmonary embolism. Thromb Haemost.

[13] Ortel TL, Neumann I, Ageno W (2020). American Society of Hematology 2020 guidelines for management of venous thromboembolism: treatment of deep vein thrombosis and pulmonary embolism. Blood Adv.

[14] Raja AS, Greenberg JO, Qaseem A, Denberg TD, Fitterman N, Schuur JD (2015). Evaluation of patients with suspected acute pulmonary embolism: best practice advice from the clinical guidelines committee of the American College of Physicians. Ann Intern Med.

[15] Konstantinides SV, Meyer G, Becattini C (2019). 2019 ESC Guidelines for the diagnosis and management of acute pulmonary embolism developed in collaboration with the European Respiratory Society (ERS): The Task Force for the diagnosis and management of acute pulmonary embolism of the European Society of Cardiology (ESC). Eur Respir J.

[16] Tosetto A, Iorio A, Marcucci M (2012). Predicting disease recurrence in patients with previous unprovoked venous thromboembolism: a proposed prediction score (DASH). J Thromb Haemost.

